# Prevalence of cardiovascular autonomic neuropathy and gastroparesis symptoms among patients with type 2 diabetes who attend a primary health care center

**DOI:** 10.1371/journal.pone.0209500

**Published:** 2018-12-21

**Authors:** Lina A. AlOlaiwi, Turki J. AlHarbi, Ayla M. Tourkmani

**Affiliations:** Family and Community Medicine Department, Prince Sultan Military Medical City, Riyadh, Saudi Arabia; Weill Cornell Medicine-Qatar, QATAR

## Abstract

**Introduction:**

Cardiovascular autonomic neuropathy (CAN) and gastroparesis are two types of diabetic autonomic neuropathy which could affect patients' quality of life and carry significant morbidity and mortality outcomes. The aim of this study was to estimate the prevalence and risk factors of both CAN and gastroparesis symptoms among patients with type 2 diabetes mellitus (T2DM) at primary health care level.

**Methods:**

A cross-sectional study was conducted among 400 adults with T2DM from April 1, 2017 to March 20, 2018. CAN was defined by the presence of any of the followings: resting tachycardia, orthostatic hypotension or prolonged corrected QT interval in the electrocardiogram. Gastroparesis symptoms were assessed using a validated questionnaire: the Gastroparesis Cardinal Symptom Index.

**Results:**

The mean age of study participants and disease duration were 55.26 ± 10.65 years and 10.77 ± 6.89 years, respectively. CAN was present in 15.3% of the participants. Hypertension, smoking, antihypertensive use, body mass index, dyslipidemia and albuminuria were significantly higher in participants with CAN than those without CAN (p<0.05). Prolonged disease duration (p = 0.007) and hypertension (p = 0.004) were independently associated with CAN. Gastroparesis symptoms were present in 6.3% of study participants and were significantly associated with those of female gender (P<0.05). Metformin use emerged as an independent predictor of the presence of at least one symptom (p = 0.001).

**Conclusion:**

Among Saudi adults with T2DM at primary care level, the prevalence of CAN is significant and is independently related to disease duration and hypertension, indicating the importance of CAN screening, especially for those with prolonged disease duration, and the importance of controlling blood pressure in order to prevent CAN or its consequences. The prevalence of gastroparesis symptoms is 6% and is independently related to metformin use, and therefore, symptomatic screening is required to decide which patients need further evaluation.

## Introduction

Saudi Arabia was ranked as one of the top ten countries with a high prevalence of diabetes with an estimated prevalence of 16.8% in 2010 and this prevalence was estimated to be 18.9% by 2030 [1]. Recent local studies have shown that the prevalence of diabetes was 13.4% among those who were aged 15 years and older, and 23.7% to 25.4% among those who were aged 30 years and older [2–4].

One of the most important and underdiagnosed complications of diabetes is diabetic autonomic neuropathy (DAN). DAN can affect any organ system which is controlled by the autonomic nervous system [5]. Screening for signs and symptoms of DAN is recommended in the presence of microvascular complications [6].

Cardiac autonomic neuropathy (CAN) is defined as a neuropathy of the cardiovascular autonomic system in the presence of diabetes and in the absence of other etiologies [7]. It was found that patients with diabetes and CAN have a higher mortality rate than patients without CAN [8–9]. The diagnosis of CAN requires evidence of symptoms and signs. Signs of CAN include decreased heart rate variability (HRV), resting tachycardia, and postural hypotension [6]. Increasingly, it has been found that there is a relationship between CAN and prolonged corrected QT interval (QTc) in electrocardiography (ECG), and prolonged QTc can be used as a direct indication of CAN [10–14].

Lifestyle modifications, glycemic control and good control of other comorbidities may show improvement and reduce the progression of CAN [15–18].

The prevalence of CAN varies due to the variability of the diagnostic criteria and tests that were used among T2DM patients [19]. It ranges between 22.1% to 61.6% [6,20–29].

Gastroparesis is another type of DAN, which was defined by Parkman et al. [30] as "a symptomatic chronic disorder of the stomach characterized by delayed gastric emptying in the absence of mechanical obstruction". Gastroparesis has a variety of symptoms, such as abdominal discomfort, bloating, nausea, vomiting and early satiety, which could affect patients' quality of life [30]. Moreover, gastroparesis may lead to poor glycemic control and possibly to postprandial hypoglycemia in patients who are on insulin [31–33]. Clinical improvement of gastroparesis has been observed with glycemic control, dietary changes, and pharmacotherapy, so early diagnosis of this problem is crucial. Scintigraphy is the gold-standard test for measuring the rate of gastric emptying [30]. However, in addition to the radiation risk, scintigraphy requires specialized equipment [34], which can limit its use. The Gastroparesis Cardinal Symptom Index (GCSI) is a validated questionnaire which assists in assessing the presence of gastroparesis symptoms and its severity [35].

Several studies have shown that upper gastrointestinal symptoms were more severe and more common among type 2 diabetes patients than non-diabetic control groups [36–40].

However, local studies regarding the prevalence of both CAN and gastroparesis symptoms among Saudi patients with T2DM are scarce. Therefore, our aim was to estimate the prevalence and risk factors of both CAN, and the symptoms of gastroparesis among Saudi adults with type 2 diabetes at primary health care level.

## Materials and methods

This cross-sectional study was conducted at the chronic diseases center at Alwazarat Health Care Center (WHC) from 1 April, 2017 to 20 March, 2018. WHC is one of the primary health care centers at Prince Sultan Military Medical City (PSMMC), Riyadh, Saudi Arabia.

Study participants were enrolled using a simple random sampling technique; a previously randomly generated list.

All Saudi adults (older than 18 years) who had T2DM and attended the chronic diseases center at the time of the study period were included.

Participants with any of the following conditions were excluded from the study: febrile, dehydration, bedridden, neurological diseases, cardiac diseases, gastrointestinal diseases, connective tissue diseases, liver diseases, gastric surgery in the past, thyroid diseases, anemia, electrolytes abnormalities, chronic kidney disease (eGFR < 60 ml/min), currently on atypical antipsychotics or antidepressants, glucagon-like peptide-1 receptor agonist, dopaminergic medications, chemotherapeutic agents, or antiarrhythmic medications. Participants who refused to participate or who did not have laboratory tests within one week were also excluded.

Each participant was interviewed individually to obtain demographic and clinical data. Height and weight were measured for calculation of body mass index (BMI) by dividing weight in kilograms over the height squared in meters. After five minutes of sitting rest, blood pressure (BP) and heart rate were measured. A second reading was taken after five minutes, and the mean of the two readings was taken as the sitting BP reading. To check for postural hypotension, BP was measured again after standing for two to five minutes.

An ECG was performed by a single technician for each participant in the supine position, using a Philips PageWriter TC50 cardiograph for measurement of QT interval. To avoid QT dispersion (differences in QT interval between different ECG leads), QT was recorded and measured electronically in lead II in all participants [13]. All ECG records were reviewed by a single expert who was blind to the participants' medical conditions to ensure the accuracy of the measurements. QTc was calculated using *Bazzet's formula* [41]:

$$QTc=\frac{QT\;interval\;(seconds)}{\sqrt{RR\;interval\;(seconds)}}$$

Twelve-hour fasting laboratory results were reviewed from participants' electronic files. These included: fasting blood sugar (FBS), total cholesterol (TC), triglycerides (TG), high-density lipoprotein (HDL), and low-density lipoprotein (LDL). Hemoglobin A1c (HbA1c) was measured based on the Diabetes Control and Complications Trial (DCCT) reference. Using the MDRD formula, the estimated glomerular filtration rate (eGFR) was calculated [42].

Morning urine samples were taken; albumin to creatinine ratios (mg/g) were classified as normal if less than 30mg/g; microalbuminuria if from 30 to 300 mg/g, and macroalbuminuria if more than 300 mg/g and the same range of albuminuria was present over the previous six months [43].

Retinopathy screening was performed using indirect ophthalmoscope after pupillary dilation by a single ophthalmologist who was unaware of the participant's medical conditions. Results were classified as “no diabetic retinopathy” if there were no diabetic retinal changes, and “diabetic retinopathy” if there were any retinal changes related to diabetes.

The diagnosis of CAN requires specialized equipment [44], but due to limitations of its use at our primary health care center, CAN was defined in the present study by the presence of any of the following: [6, 10–14]

- Prolonged QTc in ECG (more than 0.45 seconds in males and more than 0.47 seconds in females [45]),
- Orthostatic hypotension (a drop in the systolic blood pressure (SBP) by 20 mmHg or more, or a reduction in the diastolic blood pressure (DBP) by 10 mmHg or more within two to five minutes of standing [46]), or
- Resting tachycardia (resting heart rate above 100 beats per minute (bpm) [47]).

The permission to use and translate GCSI was obtained from Mapi Research Trust (MRT), and the translation was performed according to the MRT validation process [48]. The GCSI includes nine items categorized into three sub-scales: three items for nausea/vomiting, four items for post-prandial fullness/early satiety, and two items for bloating. For each item, the severity of symptoms was rated from 0 (no symptoms) to 5 (severe symptoms). Participants were considered as having gastroparesis symptoms if the GCSI was ≥ 1.9 over the previous two weeks [35].

The approval for this study was given by the Research Ethics Committee at Prince Sultan Military Medical City. An informed consent was obtained from each participant.

The collected data were entered into Microsoft Excel 2010 and then transferred to the Statistical Package for Social Sciences, version 22 (SPSS Inc., Chicago, IL, USA) for analysis. Continuous data were presented as means ± standard deviation (SD) and were compared with categorical data using *t*–test or analysis of variance (ANOVA). Categorical data were presented as frequencies and percentages (n and %) and were analyzed using chi-squared test (*x*^2^). For all analyses, *p* value level of < 0.05 was considered to indicate statistical significance.

## Results

A total of 511 participants were enrolled in the study; however, 400 met the inclusion criteria and were included in the final analysis.

The clinical features of study participants are shown in Table 1. The mean age was 55.26 ± 10.65 years, of which 56.3% were females. Moreover, the mean disease duration and the mean HbA1c were 10.77 ± 6.89 years and 8.07 ± 1.59% respectively. Almost half of the patients had hypertension and the most frequently used hypoglycemic agent was metformin (94.5%).

**Table 1:** Clinical characteristics of the study participants (n = 400).

**Age** (years)*	55.26 ± 10.65
**Gender (male/female)^**	175(43.8)/ 225(56.3)
**Duration of diabetes**(years)*	10.77 ± 6.89
**Current smoking** **^**	41 (10.3)
**Hypertension** **^**	239(59.8)
**Anti-hypertensive** **^**	257(64.3)
**Insulin** **^**	189(47.3)
**Sulfonylurea** **^**	198 (49.5)
**Metformin^**	378 (94.5)
**DDP-4^**	153 (38.3)
**TZD^**	3 (0.8)
**Meglitinides^**	1 (0.3)
**BMI** (Kg/*m*^2^)*	32.46 ± 5.41
**FBS** (mmol/L)*	8.71 ± 3.55
**TC** (mmol/L)*	4.2 ± 0.89
**TG** (mmol/L)*	1.7 ± 0.81
**HDL** (mmol/L)*	1.15 ± 0.34
**LDL** (mmol/L)*	2.55± 0.78
**HbA1c** (%)*	8.07 ± 1.59
**Serum creatinine** (μmol/L)*	67.71 ± 18.4
**eGFR** (ml/min/1.73 m^2^)*	102.02 ± 25.10
**Resting heart rate** (bpm)*	82.12 ± 12.08
**SBP** (mmHg)*	130.33 ± 17.08
**DBP** (mmHg)*	74.52 ± 9.52
**QTc** (seconds)*	0.43 ± 0.03
**Albuminuria (normo/micro/macro)^**	298(74.5)/84(21)/10(4.5)

DPP-4 inhibitor, Dipeptidyl peptidase-4 inhibitor; TZD, Thiazolidinediones; bpm, beats per minute, BMI; Body mass index, DBP; Diastolic blood pressure, eGFR; Estimated glomerular filtration rate, FBS; Fasting blood sugar, HbA1c; Hemoglobin a1c, HDL; High-density lipoprotein, LDL; Low-density lipoprotein, QTc; Corrected QT interval, SBP; Systolic blood pressure, TC; Total cholesterol, TG; triglycerides

*Data are presented as means ± SD

^ data are presented as n(%).

Among all study participants (n = 400), a total of 331 underwent an ophthalmology examination, and diabetic retinopathy was present in 23.3% (n = 77) of them.

**Fig 1:**
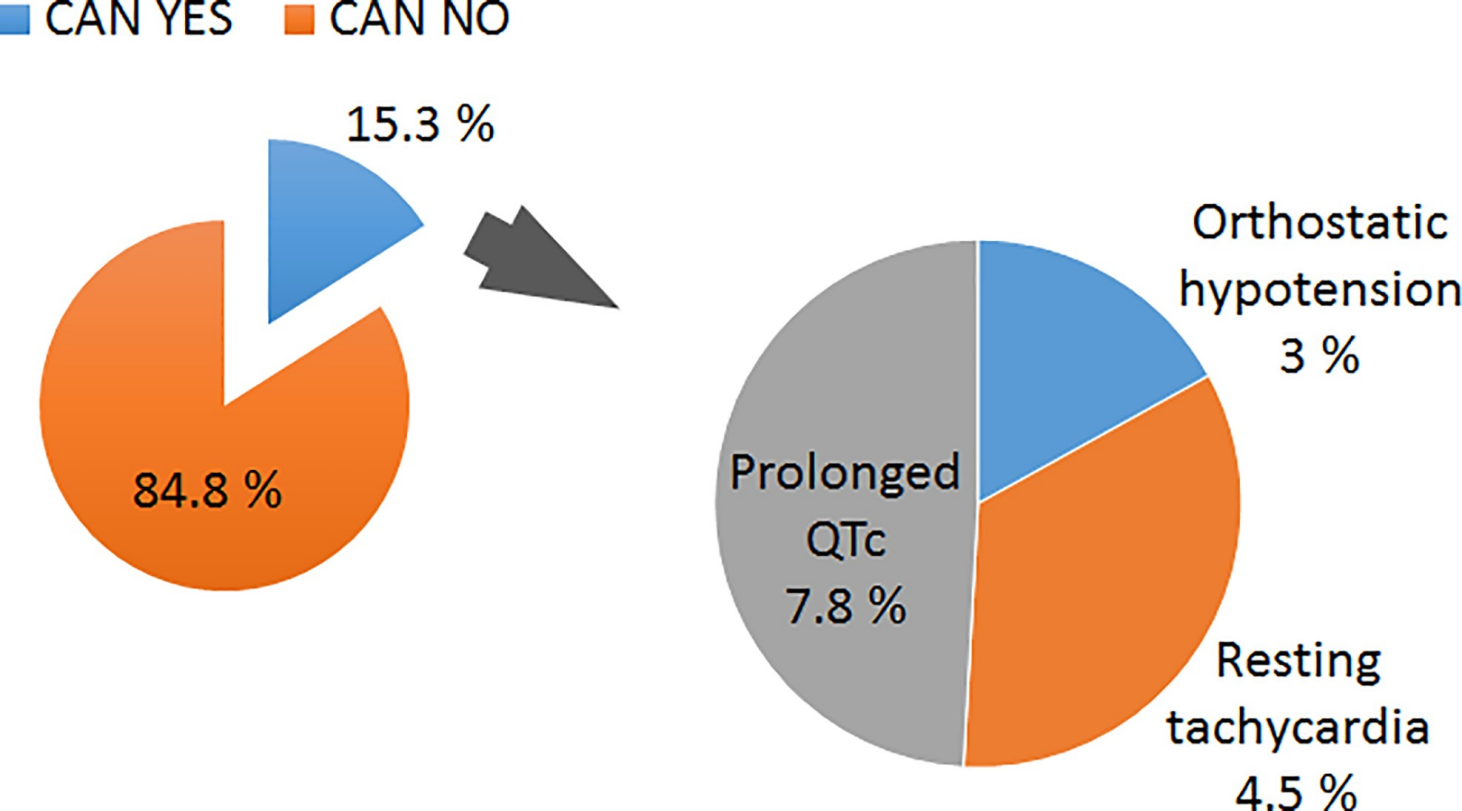
Prevalence of CAN among study participants (n = 400).

CAN was present in 15.3% of the participants (n = 61) (Fig 1).

Gastroparesis symptoms (GCSI ≥ 1.9) were present in 6.3% of participants (n = 25). The median GCSI score was 0.65 with a range of 3.2. The frequencies of individual gastroparesis symptoms are shown in Table 2, with bloating (43.8%) being the most frequently encountered symptom, followed by inability to finish a regular size meal (34.8%), and vomiting being the least encountered symptom (4.3%).

**Table 2:** Frequency of gastroparesis symptoms among study participants (n = 400).

Gastroparesis symptoms	% (n)
**Nausea**	18.3 (73)
**Retching**	10.8 (43)
**Vomiting**	4.3 (17)
**Stomach fullness**	31.8 (127)
**Not able to finish a meal**	34.8 (139)
**Excessive fullness after meals**	33.8 (135)
**Loss of appetite**	23.8 (95)
**Bloating**	43.8 (175)
**Belly visibly larger**	28.5 (114)

Table 3 summarizes the demographic and clinical variables of participants with and without CAN. Hypertension, smoking, antihypertensive use, BMI, TC, TG, BP and albuminuria were significantly higher in participants with CAN (p<0.05) compared with those without CAN. However, among those who underwent retinal screening, there was no significant association between retinopathy and CAN (p = 0.747).

**Table 3:** Demographic and clinical variables between participants with CAN and without CAN.

Variable	CAN (n = 61)	No CAN (n = 339)	*p* value~
**Male gender^**	26 (42.6)	149 (44.0)	0.889
Age (years)*	55.21± 9.84	55.26 ± 10.79	0.973
Duration of diabetes (years)*	9.18 ± 6.61	11.06 ± 6.9	0.050
**Current smoking** **^**	11 (18.0)	30 (8.8)	**0.038**
**Hypertension^**	45 (73.8)	194 (57.2)	**0.016**
**Anti-hypertension use^**	48 (78.7)	209 (61.7)	**0.013**
BMI (Kg/*m*^2^)*	34.2 ± 5.95	32.15 ± 5.25	**0.006**
SBP (mmHg)*	137.7 ± 17.48	129 ± 16.7	**0.000**
DBP (mmHg)*	77.28 ± 12.1	74 ± 8.9	**0.049**
FBS (mmol/L)*	8.93 ± 3.79	8.67 ± 3.52	0.594
HbA1c (%)*	8.47 ± 1.88	8± 1.53	0.064
TC (mmol/L)*	4.42 ± 1.03	4.16 ± 0.86	**0.035**
TG (mmol/L)*	1.99 ± 0.95	1.65 ± 0.76	**0.003**
HDL (mmol/L)*	1.1±0.27	1.16 ± 0.34	0.196
LDL (mmol/L)*	2.7 ± 0.88	2.52 ± 0.75	0.104
**eGFR (ml/min/1.73 m**^**2**^)*	104.86 ± 28.2	101.5 ± 24.5	0.338
**Abuminuria (normo/micro/macro)^**	37(61)/20(33)/ 4(6)	261(77)/ 64(19)/ 14(4)	**0.026**

BMI; Body mass index, DBP; Diastolic blood pressure, eGFR; Estimated glomerular filtration rate, FBS; Fasting blood sugar, HbA1c; Hemoglobin a1c, HDL; High-density lipoprotein, LDL; Low-density lipoprotein, SBP; Systolic blood pressure, TC; Total cholesterol, TG; Triglycerides

*Data are presented as means ± SD

^ data are presented as n(%)

~ *p-*value is significant if < 0.05

**Fig 2:**
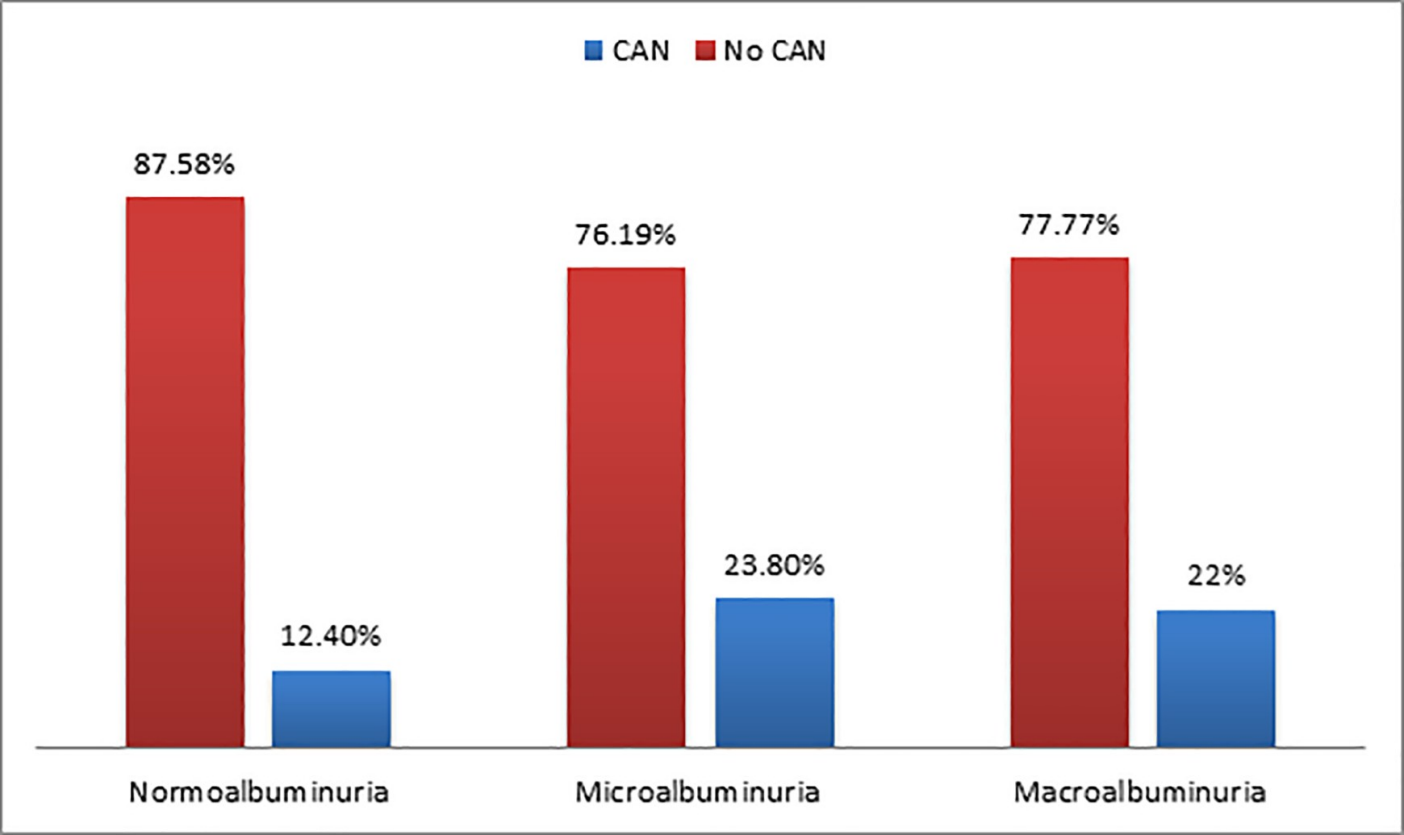
Prevalence of CAN among participants with different categories of albuminuria.

CAN was significantly higher in participants with albuminuria compared with those with normoalbuminuria (p<0.05) (Fig 2).

The clinical variables of participants with gastroparesis symptoms and those without gastroparesis symptoms are summarized in Table 4. Among all variables, female gender was significantly associated with gastroparesis symptoms. However, retinopathy was not significantly associated with gastroparesis symptoms among those who underwent retinal screening.

**Table 4:** Demographic and clinical variables between participants with gastroparesis symptoms and without gastroparesis symptoms.

Variable	Gastroparesis symptoms (n = 25)	No gastroparesis symptoms (n = 375)	*p* value~
**Gender (male/female)^**	5 (20)/ 20 (80)	170 (45.3)/ 205(54.7)	**0.020**
Age (years)*	56.44 ± 13.6	55.18 ± 10.4	0.566
Duration of DM (years)*	11.8 ± 7.6	10.7 ± 6.83	0.441
**Hypertension** **^**	14 (56)	225 (60)	0.680
BMI (Kg/*m*^2^)*	34 ± 5.68	32.36 ± 5.38	0.147
FBS (mmol/L)*	9.57 ± 4.28	8.65 ± 3.5	0.304
HbA1c (%)*	8.54 ± 1.9	8.04 ± 1.6	0.124
TC (mmol/L)*	4.37 ± 1	4.18 ± 1	0.317
TG (mmol/L)*	1.67 ± 1	1.71 ± 0.8	0.811
HDL (mmol/L)*	1.18 ± 0.4	1.15 ± 0.3	0.639
LDL (mmol/L)*	2.65 ± 0.8	2.54 ± 0.8	0.488
**eGFR (ml/min/1.73 m**^**2**^)*	109.68 ± 30.1	101.51 ± 24.7	0.115
**Albuminuria (normo/ micro/ macro)^**	18 (72)/ 7 (28)/ 0(0)	280 (75)/ 77(20)/ 18 (5)	0.398

BMI; Body mass index, eGFR; Estimated glomerular filtration rate, FBS; Fasting blood sugar, HbA1c; Hemoglobin a1c, HDL; High-density lipoprotein, LDL; Low-density lipoprotein, TC; Total cholesterol, TG; Triglycerides

*Data are presented as means ± SD

^ data are presented as n(%); ~ *p-*value is significant if < 0.05

To verify the effects of diabetes duration, hypertension, HbA1c, lipid profile and eGFR on the probability that the participants have CAN, a logistic regression was performed (Table 5). The model was statistically significant (p<0.05) and explained the 11.5% variance in CAN, and correctly classified 84.8% of cases. Increased diabetes duration was associated with an increased probability of demonstrating CAN. Participants with hypertension were 2.81 times more likely to exhibit CAN symptoms, compared with those without hypertension.

**Table 5:** Odds ratios (95% CI) of predictors of CAN among participants with T2DM (n = 400).

Independent variables	Odds ratio (95% CI)	*p* value*
**Age**	0.98 (0.95–1.02)	0.476
**Duration of diabetes**	1.07 (1.02–1.13)	**0.007**
**Hypertension**	2.81 (1.39–5.68)	**0.004**
**HbA1c**	0.86 (0.71–1.03)	0.104
**TC**	0.81 (0.45–1.47)	0.486
**TG**	0.74 (0.52–1.04)	0.087
**LDL**	1.04 (0.54–2)	0.911
**eGFR**	0.99 (0.98–1)	0.345

eGFR; Estimated Glomerular Filtration Rate, HbA1c; Hemoglobin a1c, LDL; Low-density lipoprotein, TC; Total cholesterol, TG; Triglycerides

* *p-*value is significant if < 0.05

A logistic regression was conducted (Table 6) to determine the effects of age, gender, duration of diabetes, smoking, hypertension, BMI, FBS, HbA1c, lipid profile, eGFR and metformin use on the probability that participants have at least one gastroparesis symptom. The model was statistically significant (p<0.05) and explained the 8.7% variance in gastroparesis symptoms, and correctly classified 78.3% of cases. Metformin users were 4.68 times more likely to exhibit at least one gastroparesis symptom compared with non-metformin users.

**Table 6:** Odds ratios (95% CI) of predictors of one or more gastroparesis symptom among participants with T2DM (n = 400).

Independent variables	Odds ratio (95% CI)	*p* value*
**Age**	0.99 (0.98–1.03)	0.745
**Female gender**	1.58 (0.36–1.11)	0.111
**Duration of DM**	0.98 (0.97–1.06)	0.501
**Smoking**	1.71 (0.69–4.26)	0.250
**Hypertension**	0.9 (0.51–1.59)	0.703
**Metformin**	4.68 (1.89–11.6)	**0.001**
**BMI**	0.97 (0.98–1.08)	0.301
**FBS**	0.94 (0.94–1.18)	0.372
**HbA1c**	0.92 (0.83–1.4)	0.560
**eGFR**	1 (0.99–1)	0.719

BMI; Body mass index, eGFR; Estimated Glomerular Filtration Rate, FBS; Fasting blood sugar, HbA1c; Hemoglobin a1c, HDL

* *p-*value is significant if < 0.05; lipid profile is not shown in the table.

## Discussion

The current cross-sectional study was conducted to assess the prevalence of CAN and gastroparesis symptoms in 400 T2DM patients at primary health care level. The study showed that CAN was significantly present, however, symptoms of gastroparesis were present in a minority of T2DM patients.

### 4.1 CAN

Parasympathetic dysfunction occurs early in CAN, and is usually manifested by decreased HRV and later by the presence of resting tachycardia. However, as autonomic dysfunction progresses, sympathetic fibers will be affected and orthostatic hypotension can occur [49–50]. The prevalence of CAN among T2DM patients in the present study is considered to be relatively low compared with the prevalence of 22.1% - 61.6% found in other studies. However, this difference was expected and can be explained by the use of less sensitive tests used in the present study for the detection of CAN, compared with HRV testing, which enables earlier detection and was used as a part of CAN diagnosis in other studies [6,20–29]. So, we may have missed patients with early CAN, and the actual prevalence of CAN in the present study may be higher.

In our study, QTc was prolonged in 8% of T2DM cases. It has been thought that the underlying mechanism for QTc prolongation in patients with diabetes is related mainly to sympathetic imbalance, and that parasympathetic dysregulation also has a small role [51]. Moreover, it has been discovered that the degree of QTc prolongation is correlated with the severity of CAN [25].

We found that duration of diabetes emerged as a significant independent factor for the development of CAN among T2DM patients. This finding is consistent with the literature, since several studies have demonstrated a significant association between disease duration and CAN [23,25,29, 42–43]. Additionally, it has been estimated that the annual incidence of CAN among type 2 diabetes patients is around 2% [49–50]. Moreover, the prevalence of CAN at the closeout of the DCCT study was 9% and then increased to 31% by EDIC (i.e., 13 to 14 years later) [52]. Furthermore, a cross-sectional study of 478 subjects found that the prevalence of CAN was 19.8% in subjects with prediabetes, and this increased to 32.2% in recently diagnosed T2DM patients [53].

In the present study, hypertension was also found to be a significant independent factor for the development of CAN. Several studies also showed a considerable association between CAN and blood pressure [21–23,29]. In a cross-sectional survey of 310 patients with diabetes, 172 with T2DM demonstrated that CAN was significantly associated with hypertension. Those with CAN and hypertension had a more severe form of CAN, compared with those who did not have hypertension. Moreover, CAN was discovered to be a significant independent factor for hypertension (P < 0.001) [26]. Furthermore, another cross-sectional study of 33 patients with type 2 diabetes, of whom 15 had hypertension, demonstrated that patients with type 2 diabetes and hypertension showed more impairment in cardiovascular autonomic tests, compared with those without hypertension (p < 0.05), The study concluded that hypertension has a synergistic effect with diabetes in CAN development [54].

There was a significant association between CAN and albuminuria in our study, and patients with albuminuria were more likely to have CAN than those without albuminuria. In a cross-sectional study of 132 older patients with T2DM, HRV was negatively correlated with albumin-to-creatinine ratios and was independently related to albumin-to-creatinine ratios [55]. Furthermore, several studies showed that patients with diabetes and CAN had significantly higher levels of albuminuria than those patients without CAN [21–23,29]. This association might be related to common pathophysiological mechanisms between CAN and albuminuria or the involvement of CAN in the onset or the progression of nephropathy [20,50].

A significant association between CAN and diabetic retinopathy has been observed [21–23,26,29]. However, this association was not observed in patients with newly diagnosed T2DM [56]. Similarly, the current study failed to demonstrate this association. This could be related to lack of retinopathy screening of all study participants and the relatively low prevalence of CAN in this study.

### 4.2 Gastroparesis symptoms

There are two studies that used GCSI and a cutoff point of 1.9 for assessment of gastroparesis symptoms [57–58]. The first was a study of Saudi T2DM patients with a prevalence of 10.8%, which is considered slightly higher than our prevalence. Better glycemic control in the present study might explain this difference (HbA1c level of 8.07% vs. 9.1%). Moreover, the first study was conducted at a secondary health care center, and involved more complex cases compared with our research, which was conducted at a primary health care center [57]. The second study, which was conducted among type 1 diabetic patients (T1DM), showed that the frequency of gastroparesis symptoms was 10%, which is relatively high compared with our prevalence [58]. However, this difference is expected, since T1DM patients tend to be affected by gastroparesis more frequently than T2DM patients [59].

The most frequently encountered symptoms of gastroparesis in our study were bloating, feeling excessively full after meals, and early satiety, and the least encountered symptom was vomiting. Similar findings were also observed in other studies regarding symptom frequency [34,57].

The results showed that the female gender was significantly associated with gastroparesis symptoms among T2DM patients (p = 0.020). Similarly, an Australian cross-sectional study of 1101 diabetic patients found that gastrointestinal (GI) symptoms were more common in females, and the female gender was independently associated with reporting higher numbers of GI symptoms [39]. Moreover, it has been discovered that females with T2DM tend to report more frequent and severe gastroparesis symptoms compared to males [59]. Furthermore, the female gender was found to be a significant independent predictor of gastroparesis symptoms in patients with T2DM [34,57,60]. Additionally, it has been observed that females tend to have a slower gastric emptying rate compared to males, and this difference was related to the possible effect of female sex hormones on GI motility [61–62]. The female gender has been increasingly found to be independently associated with a delay in gastric emptying time [63].

In this study, we found that metformin use emerged as an independent factor for gastroparesis symptoms in patients with T2DM. However, since The most common gastrointestinal side effects of metformin are diarrhea, nausea, abdominal pain and heartburn which were not frequently encountered in the present study, this might eliminate the possibility of metformin as a cause of GI symptoms in the current study [64–65]. Similar findings were observed by Dickman R. et al. [34] regarding metformin use and the risk of gastroparesis symptoms (OR = 1.97, 95% CI = 1.19–3.28, p < 0.05), and they related this relationship to the possibility of a synergistic effect of metformin with gastroparesis to produce more GI symptoms, or the possibility that the presence of post-prandial distress syndrome was a cause of GI symptoms, rather than delayed gastric emptying. It has been observed that acute metformin administration was significantly related to delayed gastric emptying in mice [66]. In humans, it is believed that metformin might cause delayed gastric emptying rate by probably DPP-4 inhibition [67].

The present study did not show any significant association between gastroparesis symptoms and diabetic control or disease duration. This might be explained by the low prevalence of gastroparesis symptoms in this study. However, there are conflicting data in the literature regarding this association in patients with diabetes [34,38–40,59,68].

### 4.3 Limitations

We acknowledge some limitations in our study. First, the cross-sectional design does not enable us to establish a causality relationship. Therefore, further prospective studies are required to confirm the association between study outcomes and other variables. Moreover, the lack of a non-diabetic control group for comparison is another limitation. Furthermore, the results of the present study can't be generalized to all primary health care centers, since it was conducted at a single primary health care center. Further multicenter studies are required to confirm that the study results can be generalized.

CAN was assessed by less sensitive tests in the present study, which might underestimate its actual prevalence; therefore further studies using more sensitive tests are required. Furthermore, antihypertensive medications were not held before the study for ethical considerations, which might also affect cardiovascular autonomic function and therefore their possible effect on the prevalence of CAN can't be excluded.

Gastroduodenoscopy was not performed in patients with positive gastroparesis symptoms, so the possibility of mechanical causes of symptoms could not be ruled out.

### 4.4 Recommendations

We recommend CAN screening for adults with T2DM, especially those with a long-standing disease. However, further studies are required to determine the time and frequency of CAN screening. Moreover, good control of blood pressure is recommended in order to prevent CAN and its consequences. Furthermore, symptomatic screening for gastroparesis is recommended to decide which patients require further evaluation, especially for females and those who are on metformin. However, further studies are required to verify the role of metformin on the rate of gastric emptying. Additionally, further studies are recommended to explore the prevalence of gastroparesis disease among Saudi adults with T2DM by measurement of gastric emptying rate.

## Conclusions

The prevalence of CAN among Saudi adults with T2DM at primary care level is significant and is independently related to disease duration and hypertension, which is shown to be a significant modifiable independent factor for CAN. Moreover, the prevalence of gastroparesis symptoms is around 6%. Symptoms are significantly associated with the female gender and are independently related to metformin use.

## Supporting information

S1 File Excel sheet- data analysis dated 25 March 2018 second edition. (XLSX)
